# Parental satisfaction and its associated factors towards neonatal intensive care unit service: a cross-sectional study

**DOI:** 10.1186/s12913-022-08645-4

**Published:** 2022-10-19

**Authors:** Yewlsew Fentie Alle, Bantigegn Akenaw, Shimelis Seid, Samuel Debas Bayable

**Affiliations:** 1 Departement of Anesthesia, College of Medicine and Health Sciences, Debre Tabor University, Debre Tabor, 272, Ethiopia; 2 Departement of Anesthesia, College of Medicine and Health Sciences, Debre Markos University, Debre Markos, Ethiopia

**Keywords:** NICU, Parents, Satisfaction, Service

## Abstract

**Background:**

Parental satisfaction is a well-established outcome indicator and tool for assessing a healthcare system’s quality, as well as input for developing strategies for providing acceptable patient care. This study aimed to assess parental satisfaction with neonatal intensive care unit service and its associated factors.

**Method:**

A cross-sectional study design was conducted on parents whose neonates were admitted to the neonatal intensive care unit at Debre Tabor Comprehensive Specialized Hospital, in North Central Ethiopia. Data were collected by adopting an EMPATHIC-N instrument during the day of neonatal discharge, after translating the English version of the instrument to the local language (Amharic). Both Bivariable and multivariable logistic analyses were done to identify factors associated with parental satisfaction with neonatal intensive care unit service. P < 0.05 with 95% CI was considered statistically significant.

**Results:**

The data analysis was done on 385 parents with a response rate of 95.06%. The overall average satisfaction of parents with neonatal intensive care unit service was 47.8% [95% CI= (43.1–52.5)]. The average parental satisfaction of neonatal intensive care unit service in the information dimension was 50.40%; in the care and treatment dimension was 36.9%, in the parental participation dimension was 50.1%, in the organization dimension was 59.0% and the professional attitude dimension was 48.6%. Gender of parents, residency, parental hospital stay, birth weight, and gestational age were factors associated with parental satisfaction.

**Conclusion:**

There was a low level of parental satisfaction with neonatal intensive care unit service. Among the dimensions of EMPATHIC-N, the lowest parental satisfaction score was in the care and treatment while the highest parental satisfaction score was in the organization dimension.

## Introduction

Neonatal intensive care units (NICUs) are areas that require careful risk management with a wide range of neonatal care services [1–3]. It necessitates high-cost and efficient critical care delivery with a multidisciplinary team approach that focuses on preventive strategies for improved outcomes [4–6]. Parental tension and emotions are high when their child is admitted to a neonatal intensive care unit (NICU) due to serious illnesses [7, 8].

Satisfaction is a belief and attitude about a specific service provision of an institution. Parental and patient satisfaction has become a well-established outcome indicator and tool for assessing a healthcare system’s quality, as well as input for developing strategies and providing accessible, sustainable, economical, as well as acceptable patient care [7, 9, 10]. Parental satisfaction reflects the balance between their expectations of ideal care and their perception of real and available care [3, 11, 12]. It is also one of the objectives and missions of every health care center that gives NICU care service [10, 11].

Parent and patient satisfaction has become an important aspect of hospital management initiatives for quality assurance and accreditation. Also, parental involvement has an important role in the delivery of high-quality care, ranging from assisting with activities of daily living to being directly involved in important health care decisions [13, 14]. However, parental participation may not always be possible, but, effective communication will reduce the effects of crises and improve parental satisfaction [11, 15].

Ethiopia has been working to enhance its healthcare delivery systems by focusing on quality healthcare service giving special attention to mothers and children. The Ethiopian Health Service Alliance for Quality has agreed that the initial priority area would be self-motivated and transparent partnerships that stimulate innovation in health care quality management and learning across all hospitals [16].

Given the scarcity of studies on parental satisfaction with NICU care in Ethiopia, as well as clinical observations of parents complaining about NICU care, it is critical to determine the level of parental satisfaction. So, this study aimed to identify the level of parental satisfaction and associated factors with NICU care service in Debre Tabor Comprehensive Specialized Hospital (DTCSH). Also, this study helps the administrators to understand the deficiencies of the hospital’s NICU services, and then propose corresponding improvement strategies.

## Methods and materials

### Study area and period

A cross-sectional study was conducted in DTCSH which is a public hospital established in 1934 and located in the South Gondar Zone of Amara Regional State of Ethiopia. It is 97 km to the southwest of Bahir Dar, the capital city of Amara Regional State. According to the 2007 census, the total population of Debre tabor town was 155,596. It has a latitude and longitude of 11051N3801’E11.8500 N 38.0170E with an elevation of 2,706 m (8878ft) above sea level. The hospital provides neonatal intensive care unit service with five separate NICU rooms. According to the hospital’s 2017 report, 1159 neonates were admitted to the NICU, but according to evidence from a chart review in 2020, 1489 neonates were admitted [17]. This study was conducted on parents of neonates who were admitted to NICU at DTCSH from November 05, 2021, to April 30, 2022.

### Inclusion and exclusion criteria

Parents whose neonate was discharged from NICU or transferred to high dependency neonatal ward, parents who can read and write or understand the Amharic language, and neonatal stay in NICU of less than three months were included in the study. Whereas, parents with neonatal death in NICU and parents with neonatal admissions of less than 24 h were excluded from the study.

### Sample size and sampling technique

The sample size was determined by using single proportion population formula taking 50% (P) of the proportion using a 95% confidence interval and 5% margin of error (d). The sample size was determined using the following formula.

$$n={\left({Z}_{\frac{a}{2}}\right)}^{2}P{(1-P)/d}^{2}$$

n= [(1.96)^2^ 0.5(1-0.5)^2^] **/** (0.05)^2^.

**N = 385**.

By considering a 5% non-respondent rate the final sample size was 405.

The data were collected from all consecutive parents who met the inclusion criteria until the intended sample size was achieved.

### Data collection instrument and procedures

The Amharic version anonymous questionnaire was used for data collection after translating the English version of the EMPATHIC-N tool by three language experts and then back to English by the other three experts to ensure that the translation was correct. The tool’s content validity was also examined and guaranteed by members of the Anesthesia department’s research committee.

The tool has been widely used to assess parental satisfaction with NICU care services, and it has strong reliability and validity, with reliability (Cronbach’s) values of the domains ranging from 0.82 to 0.95 [18–20]. There are five domains in the EMPATHIC-N tool: Information (12 questions with a six-point Likert scale); Care & Treatment (17 questions with a ten-point Likert scale); Parental Participation (8 questions with a six-point Likert scale); Organization (8 questions with a six-point Likert scale); and Professional Attitude (12 questions with a six-point Likert scale) [18, 21, 22].

The data were collected by three anesthetists on the day of neonatal discharge using an adopted Dutch instrument of Empowerment of Parents in The Intensive Care-Neonatology (EMPATHIC-N).

### Data quality assurance

To ensure the quality of data, pre-testing of the data collection tool (the questionnaire) was done on 5% of study parents from Felege Hiwot Comprehensive Specialized Hospital who were not included in the main study. The training was given to data collectors; data were collected and properly filled in the prepared format. Supervision was made throughout the data collection period to make sure the accuracy, clarity, and consistency of the collected data.

### Ethical consideration

Debre Tabor University provided ethical clearance, and each parent was given written informed consent after being briefed about the purpose study.

### Data entry and analysis

Data were cleaned, coded, and entered into Epidata version 4.2 and exported to SPSS version 23 for statistical analysis. The adopted EMPATHIC-N instrument was validated using analysis (validity, reliability, standard factor loadings, and factor analysis). Cronbach’s alpha was used to determine the reliability and validity of the tool. Explanatory factor analysis was done to test how well the measured variables represent the number of constructs and to identify relationships between the measured items. The inter-item correlation was used to assess the relationship between items on the same scale, whereas item-discriminant validity was used to assess the relationship between scales. After categorizing the overall mean parental satisfaction score, independent variables were analyzed using binary logistic regression with parental satisfaction with NICU care service. Variables from the bivariable logistic regression with a p-value of 0.2 were fitted to a multivariable logistic regression, and certain variables were included in the model with their clinical importance. Both crude odds ratio (COR) in bivariable logistic regression and adjusted odds ratio (AOR) in multivariable logistic regression with the corresponding 95% Confidence interval were calculated to show the strength of association. In multivariable logistic regression analysis, variables with a p-value of < 0.05 were considered statistically significant. The Mann–Whitney test was used to determine the influencing factors of the parental satisfaction domains, while the Hosmer–Lemeshow goodness of fit test was performed to ensure that the analysis model was appropriate.

### Operational definitions

Satisfied: parents who scored greater than or equal to the overall mean EMPATHIC-N values were considered satisfied.

Dissatisfied: parents who scored less than the overall mean EMPATHIC-N values were considered dissatisfied.

High birth weight: neonates with a birth weight of more than 400 g [23].

Normal birth weight: neonates with a birth weight range from 250 to 400 g [24].

Low birth weight: neonates with a birth weight of lower than 250 g [25].

## Results

This study was conducted on a total of 385 parents, with a 95.06% response rate. The majority of the parents were mothers 224 (58.2%), whereas 322 (83.6%) were married. Full-term newborns 325(84.4%) and those with respiratory issues 115 (29.9%) had the highest number of NICU admissions (Table 1).

**Table 1:** Socio-demographic characteristics of study participants (n = 385)

Variables	Frequency (n)	Percentage (%)
**Parental gender**		
Mother	224	58.2
Father	161	41.8
**Age**		
18–24	132	34.3
25–39	166	43.1
40 and above	87	22.6
**Marital status**		
Married	322	83.6
Not married	63	16.4
**Residency**		
Urban	187	48.6
Rural	198	51.4
**Educational level**		
No formal education	119	30.9
Elementary school	92	23.9
Secondary school	83	21.6
Collage and above	91	23.6
**Profession**		
Housewife	70	18.2
Farmer	106	27.5
Student	59	15.3
Gov’t employ	72	18.7
Private employ	78	20.3
**Parental hospital stay**		
≤ 15 days	244	63.4
> 15 days	141	36.6
**Neonatal gender**		
Male	188	48.8
Female	197	51.2
**Birth weight**		
High birth weight	83	21.5
Normal birth weight	162	42.1
Low birth weight	140	36.4
**Gestational age**		
Premature	60	15.6
Full term	325	84.4
**Neonatal hospital stay**		
≤ 15 days	235	61
> 15 days	150	39
**Admission diagnosis**		
Infection	65	16.9
Respiratory problems	115	29.9
Prematurity	59	15.3
Gastrointestinal problems	38	9.9
Jaundice	47	12.2
Neurological problems	21	5.5
Cardiological problems	26	6.8
Others	14	3.5

### Explanatory factor analysis for subscales of parental satisfaction with NICU-care service

Before assessing parental satisfaction levels, confirmatory factor analysis was used to ensure that the measured variables accurately represented the constructs. The factor correlation matrix was used in the analysis. KMO and Bartlett’s tests of sphericity were used to check the correlation of measurement variables, with a KMO value of 0.875 and Bartlett’s tests of sphericity (p = 0.00) respectively. The correlation matrix was checked for commonality extraction, and all item values were larger than 0.3. The parallel analysis determined the number of item components (factors), and two new components for parental participation, three new components for information and organization, and four new components for Care & Treatment and professional attitude of eigenvalues met the criteria, and the Varimax component correlation matrix was an appropriate model (Table 2).

**Table 2:** Factor loadings and identified components’ of EMPATHIC-N tool

Item	Factors			
	**1**	**2**	**3**	**4**
**Information**				
What level of doctor’s information do you have regarding the child’s expected health outcomes?	0.752			
How satisfied are you with the physicians’ and nurses’ information similarity?	0.704			
How are you satisfied with doctors’ and nurses’ honesty in providing information?	0.59			
How satisfied are you with daily discussions with doctors and nurses about your child’s care and treatment?	0.58			
How understandable was the information provided by the doctors and nurses?		0.763		
How satisfied were you with the correct information when the child’s physical condition deteriorated?	0.428	0.657		
How satisfied were you with the clear answers to your questions?		0.653		
How clear is the doctor’s information about the consequences of the child’s treatment?		0.533		
To what extent do you receive clear information about the examinations and tests?			0.713	
To what extent the information brochure received was complete and clear?			0.641	
How much clear information is given regarding a child’s illness?			0.626	
Level of received understandable information about the effects of the drugs?			0.606	
**Care & Treatment**				
Level of child’s comfort taken into account by the doctors and nurses?	0.826			
The extent of satisfaction During acute situations on availability of nurses to support?	0.822			
Level of team alertness to the prevention and treatment of pain of neonate?	0.797			
Level of care taken to nurses while in the incubator/bed?	0.634			0.501
Level of correct medication always administered on time?	0.631		0.402	
level of emotional support that has been provided?		0.84		
The doctors and nurses responded well to our own needs		0.809		
Transferals of care from the neonatal intensive care unit staff to colleagues in the high-care unit or pediatric ward had gone well?		0.782		
Every day we knew who of the doctors and nurses was responsible for our child.		0.695		
How closely did doctors and nurses collaborate during work?		0.578	0.466	
Level of a common goal: to provide the finest care and treatment for our child and ourselves.			0.774	
Level of physicians and nurses paid close attention to our child’s development?			0.756	
The team as a whole was concerned for our child and you.			0.698	
Our child’s requirements were met promptly			0.455	
The extent of doctors’ and nurses’ professional knowledge of what they are doing?				0.847
How satisfied are you with the doctors’ and nurses’ understanding of the child’s medical history at the time of admission?				0.765
How satisfied are you with the rapid actions taken by doctors and nurses when a child’s condition deteriorated?				0.723
**Parental Participation**				
How involved are you in making decisions about our child’s care and treatment?	0.803			
The nurses had trained us on the specific aspects of newborn care.	0.796			
We were encouraged to stay close to our children.	0.714			
Before discharge, the care for our child was once more discussed with us.	0.617			
Even during intensive procedures, we could always stay close to our child.		0.841		
The nurses stimulated us to help in the care of our child		0.837		
The nurses helped us in the bonding with our child	0.532	0.575		
We had confidence in the team		0.561		
**Organization**				
The Neonatology unit made us feel safe	0.885			
There was a warm atmosphere in the Neonatology unit without hostility	0.805			
The Neonatology unit was clean	0.733		0.404	
The unit could easily be reached by telephone		0.809		
Our child’s incubator or bed was clean		0.8		
The team worked efficiently		0.661		
There was enough space around our child’s incubator/bed			0.9	
Noise in the unit was muffled as good as possible			0.788	
**Professional Attitude**				
Our child’s health always came first for the doctors and nurses	0.799			
The team worked hygienically	0.747			
Our cultural background was taken into account	0.728			
The doctors and nurses always took time to listen to us		0.876		
We felt welcome by the team		0.804		
Despite the workload, sufficient attention was paid to our child and us by the team		0.584	0.508	
Nurses and doctors always introduced themselves by name and function			0.794	
We received sympathy from the doctors and nurses			0.786	
At our bedside, the discussion between the doctors and nurses was only about our child.	0.457		0.582	
The team respected the privacy of our children and us.				0.874
There was a pleasant atmosphere among the staff				0.743
The team showed respect for our child and us	0.519			0.609

### Reliability of items of EMPATHIC-N tool

The total EMPATHIC-N values and the five domains showed a good level of internal consistency. The five domains’ Cronbach’s values range from 0.639 to 0.791, whereas the total EMPATHIC-N Cronbach’s value was 0.904. The inter-item correlation (IIC) of the five domains had significant internal consistency (Table 3).

**Table 3:** Reliability of items of parental satisfaction with NICU-care service

Dimension	Number of Items	Χηρονβαχη α	Mean dimension score (SD)	Maximum possible dimension score	Inter-item correlation (IIC)	Item-discriminant validity (IDV)
Information	12	0.639	39.55(8.42)	72	0.22–0.48*	0.20–0.57
Care & Treatment	17	0.791	81.70(20.49)	170	0.14–0.64*	0.25–0.38
Parental Participation	8	0.755	24.86(7.79)	48	0.01–0.71*	0.37–0.54
Organization	8	0.729	25.22(7.39)	48	0.11–0.64*	0.34–0.69
Professional Attitude	12	0.694	38.106 (9.07)	72	0.13–0.56*	0.22–0.45
EMPATHIC-N tool	57	0.904	209.44(42.82)	410	c	c

*Significant value (p < 0.001), c = not computable

### Parental satisfaction level with NICU-care service

**Fig. 1:**
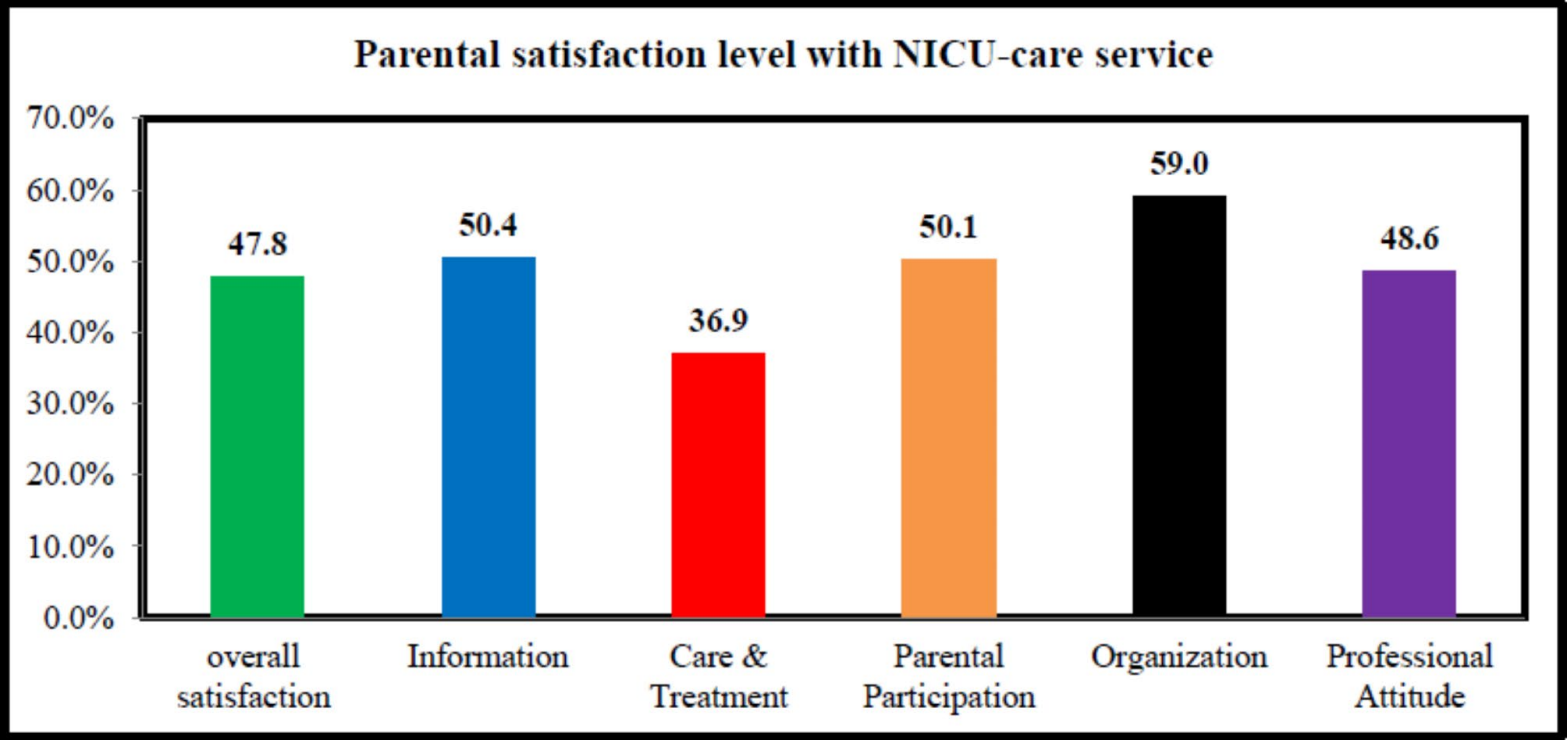
Parents’ overall and dimensional satisfaction with NICU-care services

Overall mean satisfaction level of parents in NICU-care service was 47.8% [95% CI= (43.1–52.5)]. The domains of parental satisfaction scores were compared with each other and, the lowest parental satisfaction score was for the domain of care and treatment 36.9% while the organizational domain showed the highest parental satisfaction level 59.0%. The domains of information, parental participation, and professional attitude showed comparable parental satisfaction scores (Fig. 1).

### Factors associated with the overall parental satisfaction of NICU care service

In this study, the bivariable logistic regression showed that parental gender, residency, parental hospital stay, neonatal hospital stay, birth weight, and gestational age were factors with a p-value of less than 0.2 and were fitted with a multivariable logistic regression model. However, neonatal hospital stay was not associated with multivariable logistic regression with a p-value greater than 0.05. The multivariable logistic analyses showed that mothers were 2.16 (AOR = 2.16; 95%CI: 1.28–3.63) times more satisfied than fathers. Also, parents who are from the rural area were 2.94 (AOR = 2.94; 95%CI: 1.42–6.06) more satisfied than urban. Parents who say less than 15 days were 2.18 (AOR = 2.18; 95%CI: 1.13–4.20) times more satisfied than parents who stay 15 or more days in the hospital. Also, parents of a neonate with a normal birth weight of 2.14 (AOR = 2.14; 95%CI: 1.16–3.94) and full-term neonate 2.53(AOR = 2.53; 95%CI: 1.29–4.97) times more satisfied than their counterparts (Table 4).

**Table 4:** Factors associated with satisfaction of parents in NICU-care service (n = 385)

Variables	Satisfaction level on NICU- service		Crude odds ratio	Adjusted odds ratio	p-value
	Satisfied	Not Satisfied	(95% CI)	(95% CI)	
**Gender**					
Mother	117(52.2%)	107(47.8%)	1.53(1.02,2.31)	2.16(1.28,3.63)	0.004*
Father	67(41.6%)	94(58.4%)	1		
**Residency**					
Urban	68(36.4%)	119(63.6%)	1		
Rural	116(58.6%)	82(41.4%)	2.48(1.64,3.73)	2.94(1.42,6.06)	0.004*
**Parental hospital stay**					
≤ 15 days	140(57.4%)	104(42.6%)	2.97(1.92,4.59)	2.18(1.13,4.20)	0.019*
> 15days	44(31.2%)	97(68.8%)	1		
**Birth weight**					
High birth weight	34(41.0%)	49(59.0%)	1		
Normal birth weight	96(59.3%)	66(40.7%)	2.09(1.22,3.59)	2.14(1.16,3.94)	0.015*
Low birth weight	54(38.6%)	86(61.4%)	0.91(0.52,1.58)	0.76(0.41,1.41)	0.38
**Gestational age**					
Premature	18(30.0%)	42(70.0%)	1		
Full term	166(51.1%)	159(48.9%)	2.44(1.35,4.41)	2.53(1.29,4.97)	0.007*

*= p-value < 0.05 1 = reference BWt-birth weight

Note: p-values were extracted from the multivariate logistic regression model

## Discussion

In this study, the total average parental satisfaction score with NICU service was 47.8%. This finding is nearly similar to a study done in Ethiopia (50%) [26]. Contrary to this finding, studies done in Norway (76%) [7] and Greece (99%) [27] had a higher parental satisfaction score with NICU service.

The study found that parental satisfaction with NICU service was 50.4% in the information subscale, 36.9% in the care and treatment subscale, 50.1% in the parental participation subscale, 59.0% in the organization subscale, and 48.6% in the professional attitude subscale. Parental satisfaction was lowest in the care and treatment subscale. A study in Italy [20] and South Africa [28] on the other hand, discovered the lowest parental satisfaction score in the professional attitude and parental participation subscales respectively. This disparity could be attributed to the NICU’s lack of professional and medical resources to treat and care for neonates in this situation.

According to this study, mothers, parents from rural regions, parents of neonates who stayed less than 15 days in the hospital, parents of neonates with normal birth weight, and parents of full-term neonates were all more satisfied with NICU services than their counterparts.

This study showed that mothers were more satisfied with NICU service than fathers. Similar studies conducted in Italy [20], Israel [11], and Greece [29] also showed that mothers were more satisfied than fathers in NICU service. The possible explanation might be women are allowed to spend more time in the NICU and participate in the care of their newborns and cultivate more relationships with medical caregivers than fathers.

Also in this study parents who came from rural areas were more satisfied than parents from urban areas. This founding was also similar to a study done in Greece [30] found that parents from rural areas were more satisfied. The probable reason for this might be parents from rural areas may have a low-level awareness of the hospital, expectations, and demand for NICU service as compared with actual practice.

Parents of normal birth weight and full-term neonates were more satisfied with NICU service than parents of low birth weight and preterm neonates in this study. In addition, a Norwegian study [7] found that parents of newborns with normal birth weights were more satisfied than those with low birth weights. Reasonably, parents of full-term and normal-weight neonates are likely to face unexpected concerns, which may lead to greater satisfaction with positive outcomes.

In this study, parents of neonates who stayed less than 15 days in the hospital were more satisfied with NICU service than parents who stayed 15 or more days. Similarly, studies done in Ethiopia [26] and Iran [10] found parents who stayed for a short period in the NICU were more satisfied. The possible reason for this might be that parents with short-stay are less likely to see their neonatal serious conditions that make emotional and care mismanagements.

### Limitations of the study

The study’s limitations include being a single-center nature and the lack of analysis of various alternatives to logistic regression that might be more applicable for this investigation.

## Conclusion

There was a low level of parental satisfaction with neonatal intensive care unit service. Among the dimensions of EMPATHIC-N, the lowest parental satisfaction score was in the care and treatment while the highest parental satisfaction score was in the organization dimension. As a result, health professionals and hospital administrators should collaborate to improve NICU services to provide high-quality service and satisfy parents.

## Data Availability

The datasets used and/or analyzed during the current study are available from the corresponding author on reasonable request.

## Abbreviations

AOR: Adjusted Odds Ratio
CI: Confidence Interval
COR: Crude Odds Ratio
DTCSH: Debre Tabor Comprehensive Specialized Hospital
EMPATHIC-N: Empowerment of Parents in The Intensive Care Neonatology
NICU: Neonatal Intensive Care Unit
SPSS: Statistical Package for the Social Sciences
